# A Case-Control Study Estimating Accident Risk for Alcohol, Medicines and Illegal Drugs

**DOI:** 10.1371/journal.pone.0043496

**Published:** 2012-08-28

**Authors:** Kim Paula Colette Kuypers, Sara-Ann Legrand, Johannes Gerardus Ramaekers, Alain Gaston Verstraete

**Affiliations:** 1 Department of Neuropsychology & Psychopharmacology, Faculty of Psychology and Neuroscience, Maastricht University, Maastricht, The Netherlands; 2 Department of Clinical Chemistry, Microbiology and Immunology, Ghent University, Ghent, Belgium; The Scripps Research Institute, United States of America

## Abstract

**Background:**

The aim of the present study was to assess the risk of having a traffic accident after using alcohol, single drugs, or a combination, and to determine the concentrations at which this risk is significantly increased.

**Methods:**

A population-based case-control study was carried out, collecting whole blood samples of both cases and controls, in which a number of drugs were detected. The risk of having an accident when under the influence of drugs was estimated using logistic regression adjusting for gender, age and time period of accident (cases)/sampling (controls). The main outcome measures were odds ratio (OR) for accident risk associated with single and multiple drug use. In total, 337 cases (negative: 176; positive: 161) and 2726 controls (negative: 2425; positive: 301) were included in the study.

**Results:**

Main findings were that 1) alcohol in general (all the concentrations together) caused an elevated crash risk; 2) cannabis in general also caused an increase in accident risk; at a cut-off of 2 ng/mL THC the risk of having an accident was four times the risk associated with the lowest THC concentrations; 3) when ranking the adjusted OR from lowest to highest risk, alcohol alone or in combination with other drugs was related to a very elevated crash risk, with the highest risk for stimulants combined with sedatives.

**Conclusion:**

The study demonstrated a concentration-dependent crash risk for THC positive drivers. Alcohol and alcohol-drug combinations are by far the most prevalent substances in drivers and subsequently pose the largest risk in traffic, both in terms of risk and scope.

## Introduction

Alcohol and cannabis are amongst the substances most widely used by drivers in Europe [1], [2], [3]. A recent roadside study in 13 European countries revealed that alcohol was the most prevalent (3.48%) among drivers, followed by illicit drugs (1.9%, with cannabis most frequently detected), and medicinal drugs (1.36%) [1]. Legitimate concern exists about the influence of alcohol and other drugs on traffic safety. By means of epidemiological studies, the risk of having an accident (risk assessment; case-control studies) and the risk of being responsible for a crash (responsibility or culpability estimates; also called case-crossover studies) can be calculated. Both are valid methods to study and understand the impact of drug use on traffic safety. Culpability studies have been conducted in larger numbers than case-control studies, which are scarcer, probably due to their high costs and complex logistics [4], [5].

Culpability studies focus on drivers involved in traffic accidents and classify each case according to the driver’s responsibility (yes/no) for the accident and the presence of drugs (positive/negative). These data are then taken to calculate the culpability risk for drivers under the influence of drugs. The assumption is that driving under the influence increases the risk of being responsible for causing a traffic crash [2], [6]. In general, culpability studies have indicated that alcohol as well as alcohol-drug and drug-drug combinations are associated with significantly elevated risks [5].

Case-control studies compare prevalence of drug use among drivers involved in crashes (i.e. cases) and among a control group that was not involved in traffic accidents. The assumption is that drugs that cause driver impairment will be more prevalent in cases than in controls, which can be expressed as an OR of crash risk. Ideally the control group consists of a random sample of drivers from the general driving population, but alternative approaches have been used. Mura and colleagues (2003) did not randomly select drivers as controls but instead used non-traffic involved patients with a valid driver licence [7]. A potential shortcoming of this approach is that the controls might not be a correct representation of the driving population. A population-based case-control study on the other hand gathers information from the general driving population (e.g. by means of roadside testing), and from fatally and/or non-fatally injured drivers. Both populations are screened for alcohol and drugs. Using these data, OR are calculated to estimate the accident risk associated with a specific (or combination of) substance(s) [2], [4], [6], [8]. This kind of design is preferable over the non-random selection of controls as it reflects the true driving population but only a limited number of such studies have been conducted in injured and killed drivers [4], [9], [10]. The present study is a population-based case-control study in which controls were randomly selected drivers in the same regions where the accidents happened.

Another potential shortcoming in previously conducted studies has been the use of different matrices to determine drug presence in cases and controls. Ideally, drug presence is determined in the same matrix, preferably blood, in order to maximize comparability. Comparisons of drug concentrations in distinct matrices are troublesome due to a lack of reliable transfer functions [11], [12], differences in detection windows, or due to the inability to measure the parent compound in some matrices [4]. Unfortunately, most case-control studies used different matrices in cases and controls, and compared blood data collected in cases to saliva [9] or urine [13] data from controls (review: [10]). As a consequence, many case-control studies have not been able to determine concentration-effect relations between drug use and crash risk [7]. In order to overcome this difficulty, the present study collected blood samples in both cases and controls.

The data presented in this article are part of a large-scale European project called “Driving under the influence of Drugs, Alcohol, and Medicines” (DRUID; www.druid-project.eu). The main aim of the project was to assess drug prevalence in traffic and their associated risk. Likewise, the objective of the present study was to calculate the risk of having a car accident after using a drug, or a combination of different drugs. The general hypothesis was that drug use increases accident risk.

## Methods

### Setting, Data Collection, and Study Population

A population-based case-control study was conducted in Belgium from 2008 till 2010. The detailed description of the procedure has been published [14].

Cases were drivers involved in an accident, and who were hospitalized in five hospitals in Belgium (University Hospitals of Brussels, Ghent, Leuven and Liège and regional hospital of Namur). These hospitals were involved in the Belgium Toxicology and Trauma Study of 1995 [15]. A total of 1078 blood samples from injured drivers were collected and delivered to the laboratory with the corresponding patient form. Medical staff was in charge of filling out a patient form for each participant. The patient form included the minimum required data, i.e.: Maximum Abbreviated Injury Scale (MAIS); identification number; hospital; date and time of the accident; vehicle type; drugs and fluids administered in hospital before sampling; age and gender of patient; time between accident and sampling; single or multiple vehicle accident.

Controls were a random sample of drivers on Belgian roads (roadside survey), conducted in five regions corresponding to the catchment areas of the hospitals. The procedure of this roadside survey consisted of two independent phases. The first was a random alcohol control performed by the police. The police officers were asked not to pay attention to external signs of impairment but to stop drivers at random and to test all stopped drivers. After the police procedure (amongst other: alcohol test with breathalyser), the stopped drivers were asked if they wanted to participate in the present research. If they refused, a refusal form with demographic data was filled in to be able to calculate a response rate. The second phase was the blood sample collection, which took place in a mobile research unit. The drivers were informed about the objective and the content of the research, signed an informed consent form, and were asked to fill in a questionnaire, to give a saliva sample and a blood sample, which was taken by a nurse. The questionnaire asked for the following information: type of vehicle, gender, age, education level, the result of the breathalyser test, drug control or other observations made by the police, and self report about drug, alcohol, and medicine use. Respondents who participated in the study were given a gift voucher of €20.

In total, 6163 drivers were stopped by the police. Fifty-two percent of them refused to participate, 48% agreed. The majority of respondents agreed to give both a blood and a saliva sample (93.13%), 6.73% gave only a saliva sample, 0.14% only a blood sample. In the present study, only respondents who provided a blood sample (93.27%) were included.

The study protocols of both studies were approved by the ethics committee of Ghent University Hospital. All subjects gave written informed consent. The toxicological and patient data were separated from the clinical files and made anonymous in order to guarantee the privacy of the patients.

Based on a number of criteria, cases and controls were selected from the original sample to be included in the present study. These criteria were: only car & van (as controls were restricted to those two types of vehicles) and Maximum Abbreviated Injury Scale (MAIS) ≥2, as a MAIS below 2 indicates only minor injury and we were interested in more serious injuries [16], [17]. The time between accident and sampling was never longer than 4 hours. Negative cases and controls were negative for all tested substances in blood.

**Figure 1:**
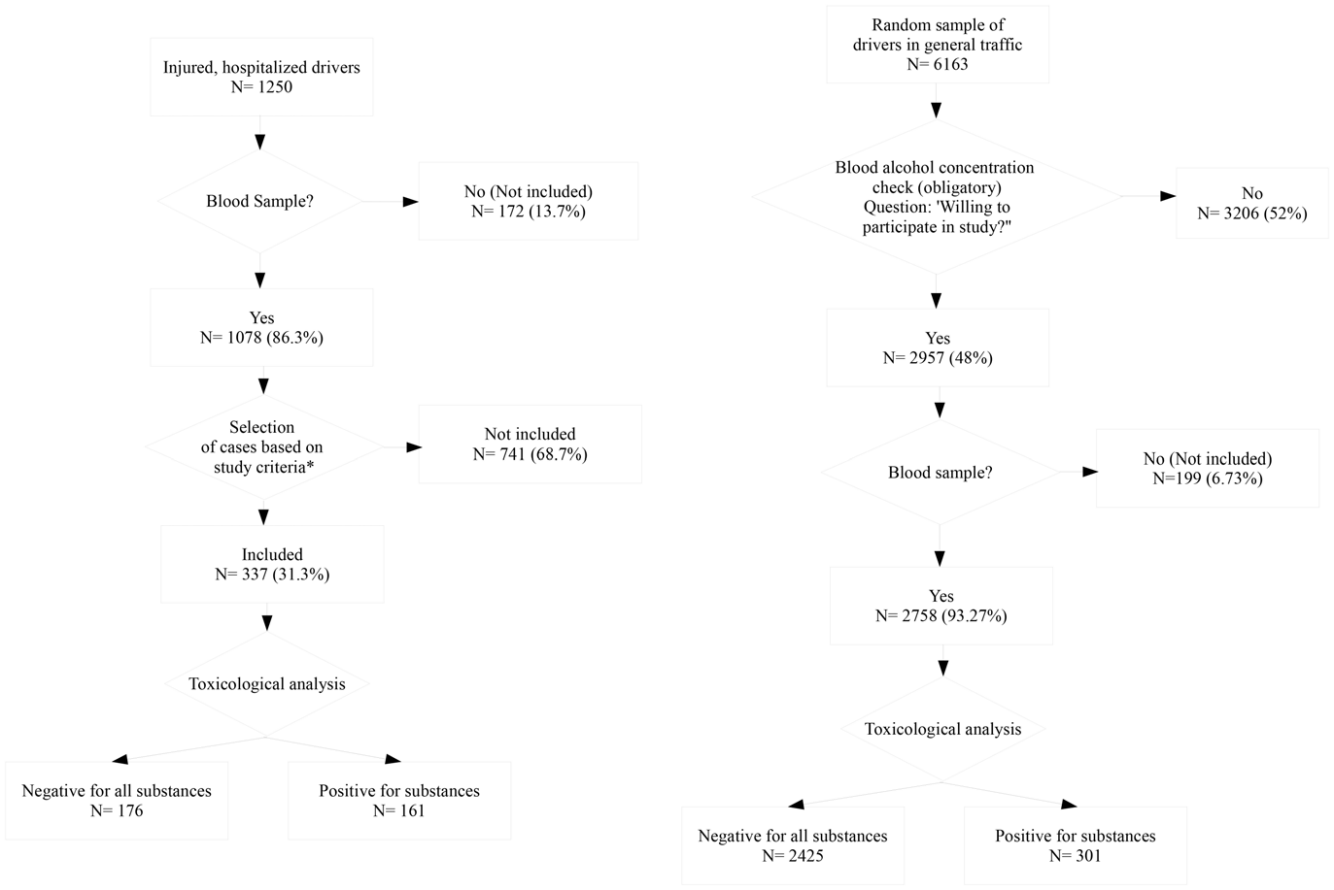
Flow chart depicting the selection procedure for the cases (left) and controls (right) included in the present study. Study selection criteria for cases: MAIS ≥2; Type of vehicle (car/van); Time between accident and sampling (<4 h).

Based on the above-mentioned selection criteria, 337 cases (negative: 176; positive: 161) and 2726 controls (negative: 2425; positive: 301) were included in the study (Fig. 1; Table 1). In both groups, the prevalence of alcohol was the highest, followed by the combination of alcohol and sedatives for the cases and benzodiazepines for the controls.

**Table 1:** Characteristics of drivers who tested positive for one drug, depicted in numbers and percentage.

Drug-only categories	Negative for all subst^s^		Alcohol		Cannabis		Benzodiazepines		Z-drugs		Medicinal Opiates	
Variable	Cases	*Controls*	Cases	*Controls*	Cases	*Controls*	Cases	*Controls*	Cases	*Controls*	Cases	*Controls*
Substance in blood												
Positive	–	–	**99**	*203*	**5**	*9*	**5**	*46*	**3**	*6*	**5**	*21*
Negative	**176**	*2425*	**–**	*–*	**–**	*–*	**–**	*–*	**–**	*–*	**–**	*–*
Gender												
Missing	**1 (0.6)**	*1 (0.04)*	**–**	*–*	**–**	*–*	**–**	*–*	**–**	*–*	**–**	*–*
Male	**104 (59.1)**	*1610 (66.4)*	**81 (81.8)**	*161 (79.3)*	**4 (80)**	*9 (100)*	**2 (40)**	*25 (54.3)*	**–**	*3 (50)*	**3 (60)**	*9 (42.9)*
Female	**71 (40.3)**	*814 (33.6)*	**18 (18.2)**	*42 (20.7)*	**1 (20)**	*–*	**3 (60)**	*21 (45.7)*	**3 (100)**	*3 (50)*	**2 (40)**	*12 (57.1)*
Age in yrs												
Missing	**4 (2.3)**	*15 (0.6)*	**2 (2.0)**	*2 (1.0)*	–	*–*	–	*–*	**1 (33.3)**	*–*	**1 (20)**	*1 (4.8)*
18–24	**31 (17.6)**	*266 (10.9)*	**19 (19.2)**	*19 (9.3)*	**3 (60)**	*6 (66.6)*	**–**	*–*	**–**	*–*	**–**	*2 (9.5)*
25–34	**50 (28.4)**	*508 (20.9)*	**34 (34.3)**	*35 (17.2)*	**2 (40)**	*2 (22.2)*	–	*3 (6.6)*	**–**	*–*	**2 (40)**	*–*
35–49	**39 (22.1)**	*929 (38.3)*	**29 (29.3)**	*70 (34.5)*	**–**	*1 (11.1)*	**1 (20)**	*19 (41.3)*	**2 (66.6)**	*2 (33.3)*	**1 (20)**	*11 (52.3)*
50+	**52 (29.5)**	*707 (29.1)*	**15 (15.1)**	*77 (37.9)*	**–**	*–*	**4 (80)**	*24 (52.1)*	**–**	*4 (66.6)*	**1 (20)**	*7 (33.3)*
Time period												
Missing	**4 (2.3)**	*–*	**2 (2.0)**	*–*	**–**	*–*	**–**	*–*	**1 (33.3)**	*–*	**–**	*–*
Week 04:00–9:59	**30 (17.0)**	*245 (10.1)*	**9 (9.1)**	*6 (3)*	**2 (40)**	*–*	**–**	*10 (21.7)*	**–**	*1 (16.7)*	**–**	*–*
Week 10:00–15:59	**46 (26.1)**	*652 (26.9)*	**4 (4.0)**	*27 (13.3)*	**1 (20)**	*3 (33.3)*	**3 (60)**	*14 (30.4)*	**–**	*3 (50)*	**2 (40)**	*12 (57.1)*
Week 16:00–21:59	**24 (13.6)**	*539 (22.2)*	**5 (5.1)**	*34 (16.7)*	**1 (20)**	*–*	**–**	*4 (8.7)*	**–**	*1 (16.7)*	**2 (40)**	*6 (28.6)*
Week 22: 00–03:59	**16 (9.1)**	*66 (2.7)*	**23 (23.2)**	*17 (8.4)*	**–**	*–*	**–**	*1 (2.2)*	**2 (66.7)**	*–*	**–**	*–*
Weekend 04:00–9:59	**12 (6.8)**	*143 (5.9)*	**14 (14.1)**	*14 (6.9)*	**–**	*1 (11.1)*	**1 (20)**	*1 (2.2)*	**–**	*–*	**–**	*–*
Weekend 10:00–15:59	**12 (6.8)**	*193 (8.0)*	**7 (7.1)**	*18 (8.9)*	**–**	*–*	**–**	*7 (15.2)*	**–**	*–*	**–**	*1 (4.8)*
Weekend 16:00–21:59	**17 (9.7)**	*403 (16.6)*	**11 (11.1)**	*49 (24.1)*	**–**	*2 (22.2)*	**1 (20)**	*7 (15.2)*	**–**	*1 (16.7)*	**1 (20)**	*1 (4.8)*
Weekend 22:00–03:59	**15 (8.5)**	*184 (7.6)*	**24 (24.2)**	*38 (18.7)*	**1 (20)**	*3 (33.3)*	**–**	*2 (4.3)*	**–**	*–*	**–**	*1 (4.8)*
Totals without missings	**168**	*2409*	**95**	*201*	**5**	*9*	**5**	*46*	**2**	*6*	**4**	*20*

Drug–only categories	Negative for all subst^s^		Alcohol+Sedatives		Alcohol+Stimulants		MultipleStimulants		MultipleSedatives		Stimulants+Sedatives	
Variable	Cases	*Controls*	Cases	*Controls*	Cases	*Controls*	Cases	*Controls*	Cases	*Controls*	Cases	*Controls*
Substance in blood												
Positive	–	–	**28**	*7*	**6**	*3*	**0**	*0*	**3**	*5*	**5**	*1*
Negative	**176**	*2425*	**–**	*–*	**–**	*–*	**–**	*–*	**–**	*–*	**–**	*–*
Gender												
Missing	**1 (0.6)**	*1 (0.04)*	**–**	*–*	**–**	*–*	**–**	*–*	**–**	*–*	**–**	*–*
Male	**104 (59.1)**	*1610 (66.4)*	**22 (78.6)**	*6 (85.7)*	**6 (100)**	*3 (100)*	**–**	*–*	**3 (100)**	*4 (80)*	**4 (80)**	*1 (100)*
Female	**71 (40.3)**	*814 (33.6)*	**6 (21.4)**	*1 (14.3)*	**–**	*–*	**–**	*–*	**–**	*1 (20)*	**1 (20)**	*–*
Age in yrs												
Missing	**4 (2.3)**	*15 (0.6)*	**4 (14.3)**	*–*	**1 (16.7)**	*–*	–	*–*	**–**	*–*	**–**	*–*
18–24	**31 (17.6)**	*266 (10.9)*	**9 (32.1)**	*1 (14.3)*	**2 (33.3)**	*2 (66.6)*	**–**	*–*	**–**	*–*	**–**	*–*
25–34	**50 (28.4)**	*508 (20.9)*	**5 (17.8)**	*1 (14.3)*	**2 (33.3)**	*1 (33.3)*	–	*–*	**2 (66.7)**	*–*	**4 (80)**	*1 (100)*
35–49	**39 (22.1)**	*929 (38.3)*	**8 (28.6)**	*–*	**1 (16.7)**	*–*	**–**	*–*	**1 (33.3)**	*–*	**–**	*–*
50+	**52 (29.5)**	*707 (29.1)*	**2 (7.1)**	*5 (71.4)*	**–**	*–*	**–**	*–*	**–**	*5 (100)*	**1 (20)**	*–*
Time period												
Missing	**4 (2.3)**	*–*	**1 (3.6)**	*–*	**–**	*–*	**–**	*–*	**–**	*–*	**–**	*–*
Week 04:00–9:59	**30 (17.0)**	*245 (10.1)*	**3 (10.7)**	*–*	**1 (16.7)**	*–*	**–**	*–*	**1 (33.3)**	*–*	**1 (20)**	*–*
Week 10:00–15:59	**46 (26.1)**	*652 (26.9)*	**1 (3.6)**	*2 (28.6)*	**–**	*–*	**–**	*–*	**2 (66.7)**	*3 (60)*	**–**	*–*
Week 16:00–21:59	**24 (13.6)**	*539 (22.2)*	**4 (14.3)**	*–*	**–**	*–*	**–**	*–*	**–**	*–*	**–**	*–*
Week 22: 00–03:59	**16 (9.1)**	*66 (2.7)*	**2 (7.1)**	*–*	**1 (16.7)**	*–*	**–**	*–*	**–**	*–*	**–**	*–*
Weekend 04:00–9:59	**12 (6.8)**	*143 (5.9)*	**2 (7.1)**	*–*	**2 (33.3)**	*3 (100)*	**–**	*–*	**–**	*–*	**–**	*–*
Weekend 10:00–15:59	**12 (6.8)**	*193 (8.0)*	**1 (3.6)**	*1 (14.3)*	**1 (16.7)**	*–*	**–**	*–*	**–**	*–*	**1 (20)**	*–*
Weekend 16:00–21:59	**17 (9.7)**	*403 (16.6)*	**6 (21.4)**	*2 (28.6)*	**–**	*–*	**–**	*–*	**–**	*2 (40)*	**1 (20)**	*–*
Weekend 22:00–03:59	**15 (8.5)**	*184 (7.6)*	**8 (28.6)**	*2 (28.6)*	**1 (16.7)**	*–*	**–**	*–*	**–**	*–*	**2 (40)**	*1 (100)*
Totals without missings	**168**	*2409*	**24**	*7*	**5**	*3*	**–**	*–*	**3**	*5*	**5**	*1*

### Toxicological Analysis of Blood Samples

Twenty-six substances were determined in whole blood and grouped into drug categories (table 2). In case of metabolites, these were always accompanied by the core substance in order to be found positive. The analyses were performed by validated chromatographic methods, coupled to (tandem) mass spectrometry [18], [19].

**Table 2:** Main drug categories, analytical findings & substances, and their cut-offs.

Main category	Analytical Findings	Analytical substance	Whole blood analyticalcut-offs (ng/mL)
Alcohol	Ethanol	Ethanol	0.1 g/L
Amphetamines	Amphetamine, Methamphetamine orMethamphetamine+Amphetamine, MDMA orMDMA+MDA, MDEA or MDEA+MDA, MDA	Amphetamine	20
		Methamphetamine	20
		MDMA	20
		MDA	20
		MDEA	20
Benzodiazepines	Diazepam+Nordiazapam or Diazepam+Oxazepam orDiazepam+Nordiazepam+Oxazepam, Nordiazapam orNordiazepam+Oxazepam, Oxazepam, Lorazepam, Alprazolam,Flunitrazepam or Flunitrazepam+7- aminoflunitrazepam,Clonazepam or Clonazepam +7-aminoclonazepam	Diazepam	20
		Nordiazepam	20
		Oxazepam	50
		Lorazepam	10
		Alprazolam	10
		Flunitrazapam	2
		7-aminoflunitrazepam	2
		Clonazapam	10
		7-aminoclonazepam	10
Cannabis	THC or THC+THCCOOH	THC	1
		THCCOOH	5
Cocaine	Cocaine+Benzoylecgonine or Cocaine	Cocaine	10
		Benzoylecgonine	50
Illicit opiates	6-acetylmorphine or 6-AM+Codeine or6-AM+Morphine or 6-AM+Codeine+Morphineor (Morphine+Codeine and Morphineconcentration> = Codeine)	6-acetylmorphine (AM)	10
		Morphine	10
		Codeine	10
Medicinal opiates and opioids	Morphine, Codeine or (Codeine+Morphine and Codeineconcentration>Morphine concentration), Methadone, Tramadol	Morphine	10
		Codeine	10
		Methadone	10
		Tramadol	50
Z-drugs	Zolpidem, Zopiclone	Zolpidem	20
		Zopiclone	10

### Statistical Analysis

Statistical analyses were performed by means of PASW Statistics version 18.0. Binary logistic regression with the Backward Stepwise Likelihood Ratio method was used to calculate crude and adjusted OR. First, it uses the likelihood ratio statistic to determine which predictors form the best model. In addition, the backward stepwise method is preferable to the forward selection as the former has a lower risk of Type II error [20].

The dependent variable was accident (yes/no), and covariates were: drug (yes/no) for the crude and the adjusted OR, and also 3 extra variables for the adjusted OR: age, gender and time of week on which the accident (cases) or the roadside control (controls) took place (See Table 1). A statistically significant association between a drug and an accident is indicated by a 95% confidence interval that does not include 1. Variables that are not in the final equation are those that did not significantly predicted the outcome; i.e. accident risk. Due to the low number of cases/controls in most of the drug categories, interaction effects between drug and gender, and drug and time period, were not included in the analyses.

Before entering the analyses, the included variables were weighted. As the distribution of the study sample in the roadside study was disproportionate to the distribution of the general driving population in the eight time periods, this had to be corrected. To that end, weight factors were calculated by dividing the general distribution of traffic by time period by the distribution of sampled drivers in the same time period. The data on the general distribution of traffic was based on traffic counts of the Flemish government Agency Roads and Traffic of 2007 (http://bestuurszaken.be/AWV). The calculated weights ranged between 0.025 and 0.187 for the 8 different time periods. For the cases, this weight was set at 1, as all accidents were registered in the sampling period in the prescribed regions.

The number of cases and controls included in the calculation of the adjusted OR was less than those included in the crude OR. Some of the cases/controls were excluded because of missing demographic data (age/gender/time period) (see Table 1). For drug categories with empty cells for either positive controls or cases, only crude OR were calculated. In order to be able to calculate the risk, one observation was added to the four cells.

All the OR that are mentioned under results are adjusted ORs, except when stated otherwise. In case time period contributed significantly to the model, OR were calculated again, including the parameter week (periods 1–4) –weekend (periods 5–8) or day (periods 1–3+5–7)-night (4+8) as a covariate. For alcohol and cannabis, concentrations were grouped into 4 and 3 concentration categories respectively (alcohol: 0.1–0.5; 0.51–0.8; 0.81–1.2 and >1.2 g/L; cannabis: 1–1.99, 2–4.99, and ≥5 ng/mL). The concentration groupings of alcohol were based on the legal limits for driving under the influence of alcohol. In most countries, there is a 0.5- concentration limit, in some a 0.8-concentration limit. The same grouping has also been used in previous epidemiological and experimental studies [7], [21], [22], [23]. The concentration groupings for THC were based on experimental research showing a cut-off of 2 and 5 ng/mL to be linked with driving-related behavioural impairment [24]. ORs were calculated for the whole range of concentrations and for the concentration groups separately. The latter approach allows determining cut-offs for alcohol and cannabis at which the accident risk significantly increases.

## Results

### Single Drugs

#### Alcohol

The OR for all alcohol concentrations (0.1− >1.2 g/mL) was statistically significant (p<.001). Including alcohol concentration groups into the model revealed a breaking point at 0.8 g/L, at which the risk of having an accident significantly (p<0.001) increased (see Table 2). All the included parameters except gender contributed significantly to the models. The contribution of age to accident risk was further analyzed including 2 age groups: a ‘young’ group (18–34) and an ‘old’ group (35–50+), the latter used as reference. The crash risk was higher in the ‘young’ group (OR: 2.07; CI: 1.58–2.71). There was no significant interaction between age group and drug, indicating that the effects of age and drug use on crash risk were independent of each other. The contribution of time period to accident risk was further analyzed including two groups: week-weekend or day-night. There was a higher risk of having an accident at night (OR: 2.85; CI: 2.04–3.99; p<.001) or in the weekend (OR: 1.58; CI: 1.20–2.09; p = .001) compared to day and week, respectively.

#### Cannabis

The OR for all the THC concentrations (1− >5 ng/mL) was statistically significant (p<.001). Including cannabis group into the model revealed a THC concentration breaking point at 2 ng/mL, at which the risk of having an accident was significantly increased. All the included parameters except for age contributed significantly to the models. Further analyses for gender and time period showed that accident risk was elevated for men (OR: 1.390; CI: 1.011–1.910; p = .043), at weekends (OR: 1.42; CI: 1.01–1.99; p = .043) and at nights (OR: 3.01; CI: 1.98–4.58; p<.001), independent of drug.

#### Amphetamines

The crude OR for Amphetamines was 54.82 (p<.001) depicting an increased crash risk when under the influence of, or after having taken, amphetamines.

#### Benzoylecgonine, Cocaine, and Illicit Opiates

The crude OR for Benzoylecgonine, Cocaine and Illicit Opiates were not statistically significantly increased.

#### Benzodiazepines, Z-drugs & Medicinal Opiates

The OR for Medicinal Opiates was not statistically significant, but there was a trend, i.e. p = .056, suggesting an increase in accident risk when driving under the influence of Medicinal Opiates. Time period contributed significantly to the model, with a higher risk of accident in the weekend.

The OR for Benzodiazepines and Z-drugs could not be calculated as the parameter drug did not contribute significantly to the model, indicating that the drug did not significantly explain the variance in the dependent variable, accident.

### Multiple Drugs

The OR for the combination of sedatives or stimulants with alcohol, combinations of multiple sedatives (p = .001) and sedatives and stimulants (p = .005) were statistically significant (see Table 3). All the OR indicated an increased crash risk. All the included parameters except for age contributed significantly to the models with an increased accident risk, independent of drug, for men, at weekends, and at nights. These analyses for time period (day-night/week-weekend) were not possible for ‘sedatives and stimulants’; and for ‘alcohol and stimulants’ (week-weekend) as there were no observations in some categories.

**Table 3:** Number of weighed controls and not-weighed cases per substance & associated odds ratio.

	*Adjusted odds ratio^o^*					*Crude odds ratio*				
Substances-Groups	Cases	Controls	OR	95% CI	*p*	Cases	Controls	OR	95% CI	*p*
Negative	**168**	*2449.51*	*–*	*–*	*–*	**176**	*2466*	*–*	*–*	*–*
Single drugs										
Alcohol-overall	**95**	*175.80*	6.77	4.99–9.18	<.001	**99**	*176*	7.87	5.887–10.51	<.001
*BAC-group 1 (0.1–0.5 g/L)*	***8***	*113.47*	*0.98*	.*47–2.05*	*Ns*	***8***	*115*	.*97*	.*47–2.03*	*Ns*
*BAC-group 2 (0.5–0.8 g/L)*	***6***	*37.74*	*2.13*	.*88–5.16*	.*092*	***6***	*38*	*2.23*	.*93–5.34*	.*073*
*BAC-group 3 (0.8–1.2 g/L)*	***8***	*11.56*	*9.56*	*3.80–24.07*	*<.001*	***9***	*12*	*10.91*	*4.50–26.42*	*<.001*
*BAC-group 4 (>1.2 g/L)*	***73***	*12.03*	*76.41*	*40.05–145.80*	*<.001*	***76***	*12*	*88.52*	*47.28–165.73*	*<.001*
Amphetamines	**–**	*–*	–	–	–	**4†**	*1†*	54.82	6.09–493.12	<.001
Benzoylecgonine	**–**	*–*	–	–	–	**1†**	*5†*	6.85	0.62–75.94	Ns
Cocaine	**–**	*–*	–	–	–	**1†**	*2†*	2.74	0.32–23.59	Ns
Cannabis-overall	**5**	*5.79*	13.40	3.95–45.42	<.001	**5**	*6*	12.10	3.62–40.43	<.001
*Group 1 (1–1.99 ng/mL*	***1***	*2.40*	*6.64*	.*63–69.59*	*Ns*	***1***	*2*	*5.84*	.*56–60.48*	*Ns*
*Group 2 (2–4.99 ng/mL)*	***2***	*1.26*	*24.83*	*2.58–238.93*	.*005*	***2***	*1*	*22.24*	*2.38–207.77*	.*007*
*Group 3 (≥5 ng/mL)*	***2***	*2.13*	*14.32*	*2.03–101.13*	.*008*	***2***	*2*	*13.16*	*1.90–91.18*	.*009*
Illicit Opiates	**–**	*–*	–	–	–	**1†**	*3†*	4.57	0.47–44.15	Ns
Benzodiazepines	**5**	*52.17*	–	–	–	**5**	*52*	1.34	.53–3.40	ns
Z-Drugs	**2**	*6.52*	–	–	–	**3**	*7*	6.45	1.63–25.52	.008
Medicinal Opiates	**4**	*19.50*	2.91	0.97–8.68	.056	**4**	*20*	3.42	1.27–9.21	.015
Multiple Drugs										
Alcohol+Sedatives	**24**	*4.50*	67.19	23.91–188.84	<.001	**28**	*5*	87.19	31.85–238.69	<.001
Alcohol+Stimulants	**5**	*3.27*	20.34	4.93–83.82	<.001	**6**	*3*	25.71	6.63–99.76	<.001
Multiple Stimulants	**–**	*–*	–	–	–	**–**	*–*	–	–	–
Multiple Sedatives	**3**	*3.83*	13.70	2.95–63.66	.001	**3**	*4*	10.98	2.40–50.12	.002
Stimulants+Sedatives	**5**	.*29*	210.97	4.90–9088.71	.005	**5**	.*29*	241.51	5.7–10239.30	.004

*Legend: ns = not statistically significant; adjusted odds ratio^o^: after removal of missing data for gender, age, & time period; †: 1 observation was added to each of the four cells; Alcohol & Sedatives ( = Alcohol +: THC, Benzodiazepines, THC & Benzodiazepines, Benzodiazepines & Z-drugs, Medicinal opiates)*, Alcohol and Stimulants *( = Alcohol +: Amphetamines, Benzoylecgonine, Cocaine, Amphetamines & Cocaine)* Multiple Sedatives *(Benzodiazepine & Z-drugs, THC & Medicinal Opiates, Benzodiazepines & Medicinal Opiates)* and Stimulants & Sedatives *(Benzoylecgonine & THC, Amphetamines & Benzoylecgonine & THC, Amphetamines & Cocaine & THC, Amphetamines & THC & Benzodiazepines, Benzoylecgonine & Medicinal Opiates).*

No OR was calculated for ‘multiple stimulants’ as there were no positive cases, nor controls in this category.

## Discussion

This population-based case-control study was conducted in order to gain insight into the accident risk associated with drug use, collecting blood of both cases and controls that was analyzed for alcohol and a number of drugs.

Alcohol, independent of concentration, was related to an increase in accident risk. This finding was expected, and in line with previously reported studies (e.g. [9], [25]). We did not find a significantly increased OR for BAC concentrations between 0.5–0.8 g/L. This is in contrast with other reports [25] that showed elevated OR with this BAC range. In the present study, the OR (2.13) did approach significance (p = 0.092) indicating a trend towards increased risk. Possibly the low number of cases in this particular BAC range hampered a reliable estimation of the associated risk [26], [27]. Combined alcohol-drug use was 3 times higher in cases as compared to controls in this particular BAC range. The present data largely confirm the findings of the Grand Rapid study, and its replication in 2005 [28], [29]. Both showed a high risk for blood alcohol concentrations ≥0.8 g/L and the risk increased extremely above 1.5 g/L.

Overall, cannabis increased crash risk of drivers positive for THC. These findings fit previous culpability and case-control studies that have reported that drivers under the influence of THC are at increased risk of becoming involved in a crash [6], [25]. The THC-induced crash risk became prominent for a THC concentration >2 ng/mL. The latter seems in line with previous epidemiological reports. Laumon and colleagues (2005) showed a THC increase in culpability risk for a THC concentration >2 ng/mL [30]. Khiabani and colleagues (2006) showed that drivers who were judged to be impaired had a median THC blood concentration of 2.5 ng/mL which differed significantly from drivers that were judged to be not impaired (1.9 ng/mL) [31]. Experimental data previously also demonstrated that THC induced an increase in performance impairments at low concentrations. Serum THC concentrations between 2 and 5 ng/mL have been identified as a threshold above which THC induced impairment of skills related to driving become apparent [24].

OR for intermediate THC levels appeared higher as compared to higher levels of THC. It should be noted here that absolute OR levels are likely to be affected by the low number of cases in each THC concentration range. This was also indicated by the very wide confidence intervals associated with the OR [27]. In this context, the finding that crash risk increased in a concentration dependent manner (i.e. at low concentrations, not a significantly elevated risk, at higher concentrations an elevated risk) may be more relevant in the present study, than the absolute magnitude of the risk. The latter is likely to become more accurate with higher numbers of positive cases and controls.

The main strength of the present study was the use of the same type of biological samples which made concentration-effect relations possible. A potential weakness however was the fact that we had no positive cases/controls for a number of substances (e.g. amphetamines) and were therefore not able to calculate the (adjusted) OR. This was addressed by adding an extra observation in each of the four cells in order to calculate crude OR, i.e. not adjusting for potential confounding variables. Only amphetamines caused a statistically significant elevated crash risk. However, the accompanying confidence interval was very wide and attributable to the low number of positive cases and controls. Furthermore, the other crude OR for benzoylegonine, cocaine, and illicit opiates (for which there were no positive cases) showed elevated, but non-significant OR. The results are in line with previous findings and certainly do not show lowered risks (e.g. [25]).

A second potential limitation, interwoven with the main strength of the study, is the consequence of collection of blood samples. Due to this invasive procedure, the refusal rate or non-response rate was high amongst controls. Fifty-two percent of them refused to participate. Although these numbers are comparable to other case-control studies [4], the risk of selection bias exists. This can lead to an underestimation of the prevalence of drugs in the general driving population and an overestimation of the risk associated with particular drugs. Inspection of the demographic variables of both groups showed that there were small but significant differences between characteristics of participants and refusers. Relative to cases, males were underrepresented and females overrepresented in the control group. Likewise, participants in the age category 18–24 and 50+ were overrepresented whereas 25–34 year olds were underrepresented in the controls. For type of vehicle it was shown that vans were underrepresented. For the majority of findings reasonable explanations can be presented. For young people, the incentive (of € 20) was more attractive than for the 25–34 year olds who have a busy life and an income, and not much extra time to spend on unplanned things like the present research. The under-representation of vans in the control sample could be linked with time constraints as most of those people are on the road for their work. There were no differences in the prevalence of alcohol and the distribution in BACs among study participants and non-respondents, suggesting that the result of the alcohol test did not influence their decision to participate or not. In addition, the latter could imply that ‘drug use’ in general did not influence their decision to enter the study and agree with the sample collection or not. However, it can not be ruled out that the prevalence of medicines and drugs is higher in the control group than currently reported. Therefore, the emphasis must not be on the absolute values of the OR but on the fact that elevated crash risks are associated with the reported drugs in a concentration-related manner. Concentration-effect relations are usually not influenced by refusal rate among controls and therefore provide additional evidence for the association between drug use and crash risk.

In conclusion, the study demonstrated that THC increased crash risk in a concentration related manner. It was also shown that alcohol and alcohol-drug combinations are by far the most prevalent substances in drivers and subsequently pose the largest risk in traffic both in terms of risk and scope.
